# Is Socioeconomic Status of the Rearing Environment Causally Related to Obesity in the Offspring?

**DOI:** 10.1371/journal.pone.0027692

**Published:** 2011-11-16

**Authors:** Kevin R. Fontaine, Henry T. Robertson, Claus Holst, Renee Desmond, Albert J. Stunkard, Thorkild I. A. Sørensen, David B. Allison

**Affiliations:** 1 Division of Rheumatology, Johns Hopkins University School of Medicine, Baltimore, Maryland, United States of America; 2 Section on Statistical Genetics, Department of Biostatistics, University of Alabama at Birmingham, Birmingham, Alabama, United States of America; 3 The Institute of Preventive Medicine, Copenhagen University Hospital, University of Copenhagen, Copenhagen, Denmark; 4 Clinical Nutrition Research Center, University of Alabama at Birmingham, Birmingham, Alabama, United States of America; 5 Department of Medicine, University of Alabama at Birmingham, Birmingham, Alabama, United States of America; 6 Weight and Eating Disorders Program, Department of Psychiatry, The University of Pennsylvania, Philadelphia, Pennsylvania, United States of America; University of Hong Kong, Hong Kong

## Abstract

We attempt to elucidate whether there might be a causal connection between the socioeconomic status (SES) of the rearing environment and obesity in the offspring using data from two large-scale adoption studies: (1) The Copenhagen Adoption Study of Obesity (CASO), and (2) The Survey of Holt Adoptees and Their Families (HOLT). In CASO, the SES of both biological and adoptive parents was known, but all children were adopted. In HOLT, only the SES of the rearing parents was known, but the children could be either biological or adopted. After controlling for relevant covariates (e.g., adoptee age at measurement, adoptee age at transfer, adoptee sex) the raw (unstandardized) regression coefficients for adoptive and biological paternal SES on adoptee body mass index (BMI: kg/m^2^) in CASO were -.22 and -.23, respectively, both statistically significant (p = 0.01). Controlling for parental BMI (both adoptive and biological) reduced the coefficient for biological paternal SES by 44% (p = .034) and the coefficient for adoptive paternal SES by 1%. For HOLT, the regression coefficients for rearing parent SES were -.42 and -.25 for biological and adoptive children, respectively. Controlling for the average BMI of the rearing father and mother (i.e., mid-parental BMI) reduced the SES coefficient by 47% in their biological offspring (p≤.0001), and by 12% in their adoptive offspring (p = .09). Thus, despite the differing structures of the two adoption studies, both suggest that shared genetic diathesis and direct environmental transmission contribute about equally to the association between rearing SES and offspring BMI.

## Introduction

In its strategic plan for obesity research, the National Institutes of Health of the United States proposed, “Socioeconomic status is also related to the incidence and prevalence of obesity, such that the poor are disproportionately affected by obesity, regardless of race/ethnicity. Research is needed to further understand the impact of socioeconomic status on the development of obesity.” The inverse association observed in developed societies between obesity and indicators of socioeconomic status (SES), such as occupational status, income, and education has been well established [1]–[4] for nearly a half century [5], [6]. However, the nature and magnitude of the causal connection implied by the word “impact” in the quotation above is less clear.

There are multiple plausible causal relations underlying the SES-obesity association [7]. These include the hypotheses that: (a) lower SES causes an increased likelihood of development of obesity [8], [9]; (b) obesity causes a decline in SES through factors such as cognitive and educational difficulties [10]–[12] downward marriage, lost earnings due to sickness, or employment and wage discrimination [13]; and (c) obesity and low SES share some common genetic or environmental causes. Because both obesity [14]–[16] and SES [17]–[19] are, to some extent, under genetic control, both pleiotropic effects and correlated genetic effects may be considered, the latter possibly due to assortative mating of wealthy and thin people [20]. Thus, because obesity is genetically influenced and may lead to low SES, the observed association between low SES in the rearing environment and the development of obesity in the offspring may be only an epiphenomenon due to genetic transmission of the predisposition to obesity [21].

Although these hypotheses are neither mutually exclusive nor exhaustive, it is important to distinguish between hypothesis (a) and the others. That is, if it is true that low SES increases the likelihood of obesity through a direct causal effect then efforts to identify and subsequently modify the causative aspects of a low SES rearing environment would be justified. Alternatively, if the association is due to obesity causing a decline in SES then policies designed only to modify SES may not be effective.

Voluntary adoptions, in which young children are randomly assigned to rearing environments differing in SES, can be thought of as ‘natural experiments’ that can be used to estimate whether there are potential causal effects of the SES of the rearing environment on subsequent obesity. A Danish adoption study that implemented this notion found that the parental SES of both biological and adoptive parents was inversely associated with BMI in the adult offspring [21]. Previous analyses of these adoption data showed that the BMI of the adoptees was associated with the BMI of the biological parents, but not of the adoptive parents [14]. Under the general assumptions of typical adoption studies, if a trait in the offspring is correlated with a trait in their biological parents who did not raise them, the cause of that correlation is generally presumed to be genetic. In contrast, when a trait in the offspring is correlated with a trait in the adoptive, biologically unrelated, parent who raised them, that correlation is generally presumed to be due to environmental factors. Herein we searched the literature for relevant adoption datasets and combined analyses of parental BMI and SES data to evaluate the extent to which associations between SES of the rearing environment and offspring obesity are consistent with the notion that, besides any common genetic effects, SES *causally* contributes to obesity.

Specifically, if the SES of the rearing parents does causally affect the child's BMI and there were no other ways of generating this association, then a correlation should exist between the rearing parents' SES and the BMI of the children they raise, independent of biological parental BMI. Moreover, the magnitude of correlation should be the same whether the child is a biological or adopted offspring. In contrast, if the correlation is partly or fully due to a shared genetic diathesis between SES and BMI, then (i) the correlation between the rearing parents' SES and the child's BMI should be smaller for the adopted offspring than for the biological offspring, (ii) a correlation should exist between the SES of the biological parents and the BMI of the adopted-away offspring, and (iii) the correlation between parental SES and biological offspring BMI should be reduced in absolute value when parental BMI is controlled (see Appendix S1 which further elucidates a model underlying these expectations).

**Figure 1:**
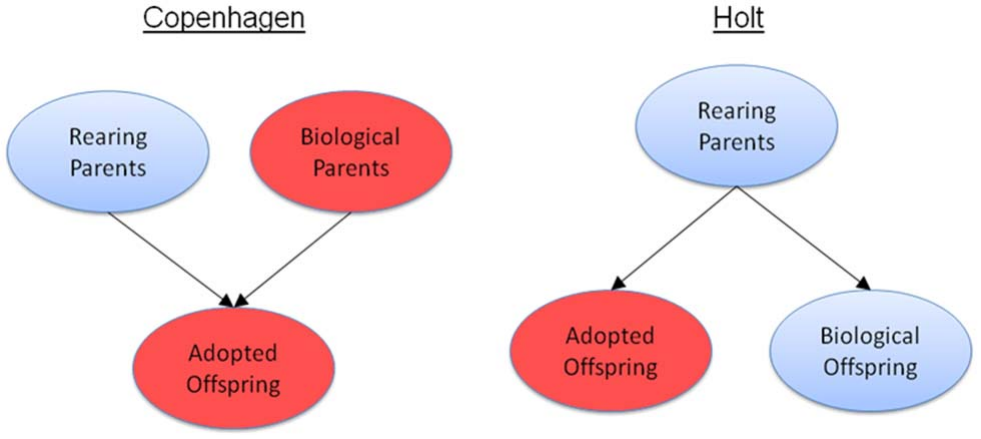
Differences in two study designs.

In summary, our primary goal was to investigate whether the association between rearing parent SES and adoptee BMI was statistically significant, and whether it remained so even after controlling for rearing parents BMI. We then sought to disentangle the respective contributions of environmental and biological components of the association of SES of the rearing environment and obesity in the offspring (Figure 1).

## Methods

### Ethics Statement

This study was declared non-human subject's research by the Institutional Review Board of the University of Alabama at Birmingham.

### Inclusion Criteria and Dataset Search Procedures

We define a family unit to be a collection of any analyzable combination of adopted offspring, biological offspring of parents in rearing household, adoptive/rearing parents, and biological parents as defined in the text. We used data from adoption studies that met the following criteria: (1) the study provides information on the weight, height, and/or BMI (measured or self-reported) of persons raised in adoptive families (adoptees); (2) the study provides information on the weight, height, and/or BMI (measured or self-reported) of either adoptive siblings (defined here as the biological offspring of the adoptive parents) or both the adoptive rearing parents and the biological parents of the adoptees; (3) the study provides information on the SES of the rearing environment (variables such as income, occupational prestige, and education); (4) the data is publicly available or readily obtainable; and (5) if data were available on the biological offspring of the adoptive parents, the regression of the adoptee BMI on their parents' SES indicators had to be negative in sign and statistically significant. The reason for including criterion #5 is because we did not wish to assess whether the SES-obesity association is present in every available dataset (the association does not necessarily exist in all populations [3], [4], [22]). Rather, we wished to study samples where such an association existed in order to investigate whether we could disentangle the various contributions of environmental and biological components to the association. In Appendix S1, we describe simulation studies which show that criterion #5 is unlikely to have induced any non-trivial bias.

To obtain data from adoption studies that met the aforementioned criteria, we searched the following electronic sources: Inter-University Consortium for Political and Social Research (http://www.icpsr.umich.edu); the National Center for Health Statistics (http://www.cdc.gov/nchs/express.htm); the Economic and Social Data Service, United Kingdom (http://www.esds.ac.uk/access/access.asp); the UK Data Archive (http://www.data-archive.ac.uk/); the Henry A. Murray Research Archive (http://www.murray.harvard.edu/); and the National Library of Medicine's Medline and pre-Medline dataset (http://www.ncbi.nlm.nih.gov). We also contacted colleagues to ask if they were aware of any adoption studies that might meet our inclusion criteria.

Our search yielded eight datasets, two of which (The Survey of Holt Adoptees and Their Families, 2005 [HOLT] and The Copenhagen Adoption Study of Obesity [CASO]) met our inclusion criteria. The 6 other datasets (the National Health Interview Survey, 1987 Adoption Supplement; the National Child Development Study, UK; the Colorado Adoption Project; the Iowa Adoption Project; the Family Life Project; and the Panel Study of Income Dynamics (PSID) Childbirth and Adoption History) did not meet all criteria (i.e., did not contain weight/height or BMI information on adoptive siblings or the adoptive rearing parents and the biological parents of the adoptees).

### Overview of Datasets Used

The two datasets used, the Copenhagen Study of Obesity (CASO) and the Survey of Holt Adoptees and Their Families (HOLT), are described below and in Tables 1 and 2. For both datasets, there was no minimum SES level required to adopt a child.

**Table 1:** Overview of the Datasets Used in the Analysis.

Characteristic	Copenhagen Adoption Study of Obesity (CASO)	Survey of Holt Adoptees and Their Families (HOLT)
Dates of Study	1924–1947	2004–2006
Adiposity Indicator	Body mass index (BMI)	Body mass index (BMI)
Socioeconomic Status (SES) Measures	Occupational prestige scores	Income and education
Country	Denmark	USA
Total number of family units in the analyses	831	1,207
Race of adoptees	European/Danish	Korean-American
Adopted Offspring (N)	831	1,690
Biological offspring of parents in rearing household (N)	0	1,196
Adoptive/Rearing parents (N)	1,637	2,414
Biological Parents (N)	1,493	0

**Table 2:** Descriptive Statistics of the Datasets Used* (before imputation).

Dataset	Variable	Adopted Offspring	Biological offspring of parents in rearing household	Adoptive/Rearing parents	Biological Parents
**CASO**	N	831	0	827 mothers 811 fathers	817 mothers 723 fathers
	Age, mean (sd)	45.1 (8.3)	N/A	33.5 (5.7) – mother 36.0 (6.5) – father	24.3 (5.5) – mother 29.3 (8.6) – father
	Sex, % female	56.4%	N/A	50%	50%
	BMI, mean (sd)	25.0 (5.5)	N/A	24.1 (4.0) – mother 25.3 (3.3) – father	23.9 (4.4) – mother 25.1 (3.6) – father
	Obesity (BMI ≥30)	21.0%	N/A	7.8% - mother 7.4% - father	7.9% - mother 8.2% - father
**HOLT**	N	1690	1196	2414	0
	Age, mean (sd)	28.2 (4.6)	32.3 (5.1)	59.6 (6.3) – mother 62.1 (7.0) -father	N/A
	Sex, % female	70.5%	37.8%	50%	N/A
	BMI, mean (sd)	23.1 (3.7)	24.0 (4.0)	25.6 (4.9) mother 27.4 (4.2) father	N/A
	Obesity (BMI ≥30)	5.8%	N/A	16.0% - mother 21.5% - father	N/A

*For CASO, because of the sampling procedure used, the sample proportions reported in this row are valid descriptors of the sample utilized, but not of the population from which the sample was drawn. In the population overall, the prevalence of obesity was roughly 4% at the time the data were collected [14].

#### The Copenhagen Study of Obesity (CASO)

Based on the Danish Adoption Register, the CASO [15] consists of non-familial adoptions in the Copenhagen area between 1924 and 1947. Around 1980, height, weight, and highest weight ever were obtained from 3,651 adoptees, among whom 831 were selected on the basis of their BMI as probands representing the extremes and the central part of the distribution. The biological and adoptive families of the proband adoptees were identified, and their weight and height were obtained by mailed questionnaires. Parent reports of height and weight were used if available. If data from parents were unavailable, due to death or some other circumstance, the information was obtained from the offspring (i.e., adoptive offspring for adoptive parents and biological offspring for biological parents). Three-hundred and ninety (36%) of the adoptive offspring provided adoptive parental data, while 208 biological offspring (19.2%) provided biological parental data. This potentially introduces uncertainty and hence dilution of the correlations, but hopefully no systematic bias [23]. Information about the occupation of the biological and adoptive fathers was available in the original adoption records for granting the adoption. The occupation was converted to a prestige-based SES score, developed in the late 1950s by Kaare Svalastoga, from 3,000 Danish occupations that were rated on a validated 8-point scale, ranging from 0 (unskilled worker) to 7 (advanced professional positions) [17]–[19]. The prestige of a given occupation associates moderately well with variables such as income and educational attainment [17]. Only paternal (both adoptive and biological) SES was used because in that time period in Denmark, far fewer women, especially of the age considered, than men would have had occupations that characterized the household's SES. The range and variability of SES is considered a reasonable representation of the general Danish population at the time the data were collected [17]. The distribution of SES values of the adoptive fathers was as follows: unskilled worker (14.5%), semiskilled worker (10.4%), skilled worker (26.5%), subordinate clerk (14.4%), skilled worker with own business (20.9%), sub-academic professions (8.5%), academic positions (4.5%), and advanced professional positions (0.3%). Though not definitive, it is possible that adoptive fathers in CASO may have a higher mean SES than the general population while adoptees have a mean SES that is comparable to the general population [17], [18]. Age at transfer of the adoptee to the adoptive family was also available in the original records. Thus, the BMI and SES of both the biological and rearing parents were fitted as predictor variables; this adjusted for any shared genetic diathesis between BMI and SES.

#### The Survey of Holt Adoptees and Their Families (HOLT)

The study focused on families who adopted a Korean-American child through Holt International Children's Services from 1970 to 1980. The adoptees were quasi-randomly assigned to families in infancy using a queuing policy (i.e., on a first come, first served basis). The agency conducted a follow-up survey, HOLT, for family members when the adoptees had grown into adulthood [24]. The HOLT was conducted from January 2004 to June 2006 and was designed to assess the health, educational attainment, and socioeconomic status of adult Korean-American adoptees and their adoptive families. Adoptive parents and a small subset of adult adoptees were surveyed, and each case represented an adopted or non-adopted child in the family. Adoptive parents provided their age, sex, marital status, occupation, education level, household income, height, weight, tobacco and alcohol usage, and the number of children they had. Adoptive parents also provided similar information on their adopted and non-adopted children. Fifty-eight percent of families had an annual household income greater than $40,000; 46.8% of adoptive mothers had 16 or more years of education; and 64.2% of fathers had 16 or more years of education. The overall survey response rate was 27%.

### Statistical Analysis

We opted to use traditional regression modeling, as opposed to structural equation modeling, for the following reasons: (1) ease of implementation, (2) ease of communication, and (3) robustness. Regarding robustness, structural equation modeling is more sensitive to violations in assumptions or normality than ordinary least squares (OLS) regression. Moreover, OLS regression also afforded us the opportunity to perform sensitivity analyses and diagnostics (i.e., bootstrapping, residuals analyses) to examine robustness. In each dataset, missing data were handled by multiple imputation [25] as described in Appendix S1.

#### The Copenhagen Adoption Study of Obesity (CASO)

The dependent variable modeled was adoptee BMI. Residuals from models with adoptee BMI as the dependent variable were not normally distributed. Hence, adoptee BMI data were normalized by a log transformation and then re-scaled to have the same mean and variance as the original data. Covariates included adoptee age at measurement, adoptee age at transfer, adoptee sex, and age of all parents (adoptive and biological) at the time of the adoptee's birth. The primary independent variables were adoptive and biological paternal SES. The difference between the regression coefficients for adoptive versus biological paternal SES were tested as described by Neter et al. [26]. Investigating this difference allows us to assess whether there is a possible causal effect of the SES of the rearing environment. Specifically, if the magnitude of the regression coefficients for the biological and the adoptive father's SES are similar and not significant, it would suggest that the genetic influence on BMI and its influence on SES of the rearing environment each accounted for roughly half the correlation between rearing environment SES and BMI. This was accomplished by using a simple linear reparametrization of the model such that one of the original two predictor variables (adoptive and biological paternal SES) are replaced in the model by their sum and the other variable is retained in its original form. Under this parameterization, the test of whether the raw (unstandardized) regression coefficient for the variable retained in its original form is not zero is arithmetically identical to a test of the equality of the regression coefficients for the two original predictor variables. Changes in parameter estimates when additional variables were included in the models were tested as described by Clogg et al. [27]. Clogg et al showed that in linear regression analysis, if a regression coefficient for a predictor (*X_1_*) is not zero in a univariate model with *Y* as a response variable and if X_1_ is also significantly correlated with another variable, X_2_, then if the coefficient for X_2_ is significant when added into a regression model also containing X_1_, this is equivalent to showing that the coefficient for X_1_ changed significantly with the inclusion of X_2_ in the model. Sensitivity analyses were conducted via bootstrap to ensure that any departure from normality due to the extreme sampling plan used by CASO (see above) did not lead to biased inference.

#### The Survey of Holt Adoptees and Their Families (HOLT)

Because data on multiple children from the same family were available, a linear mixed model (LMM) with correlated residuals in a compound symmetric structure was used to account for within-family effects. HOLT included 2,886 children from 1,207 families. The predictor variables for HOLT included the child's age and gender, the adoptive family's SES, the adoptive mother's BMI, and the adoptive father's BMI. The child's BMI was regressed on these variables. The adoptive family's SES was computed through Principal Components Analysis (PCA). The PCA incorporated the mother's education (highest completed grade), the father's education (highest completed grade), and household income. Z-scores from the standardized first principal component were used as predictors.

## Results

### The Copenhagen Adoption Study of Obesity (CASO)

In the regression of adoptee BMI on biological and adoptive paternal SES, after controlling for covariates (i.e., adoptee age at measurement, adoptee age at transfer, adoptee sex, and age of all parents (adoptive and biological) at the time of the adoptee's birth), the joint regression coefficient of adoptive and biological paternal SES (that is the value if the regression coefficient estimated for both adoptive and biological parents' SES is constrained to be equal) was statistically significant (p = 0.011). The difference between the regression coefficients for the biological and the adoptive father's SES was not significant (p = 0.982) and the two coefficients were very similar (-.22 and -.23, respectively). This supports the notion of a possible causal effect of the SES of the rearing environment in that the correlation between the genetic influence on BMI and its influence on SES each accounted for roughly half the correlation between rearing environment SES and BMI (see Appendix S1 for an elaboration of the model underlying this conclusion).

**Figure 2:**
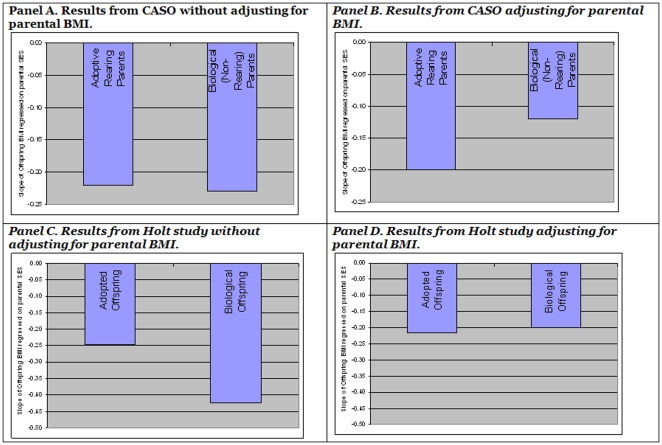
Effect of SES on biological and adopted children in the CASO and HOLT studies.

Controlling for parental BMI (both adoptive and biological) reduced the coefficient for biological paternal SES by roughly half (reduced by 44% in absolute value). Given that biological parent BMI is significantly correlated with both parental SES and with adoptee BMI, this reduction was statistically significant (p = 0.034; cf. 25). In contrast, controlling for parental BMI (both adoptive and biological) hardly changed the coefficient for adoptive paternal SES at all (reduced by 0.7% in absolute value). This is consistent with the idea that controlling for biological parental BMI controls for the correlated genetic diathesis, but not for any causal effect from SES (See Figure 2, panels A & B).

### The Survey of Holt Adoptees and Their Families (HOLT)

The rearing parents' BMI was positively associated with the BMI of biological children, but not with adopted children, which is consistent with a genetic influence on BMI [12]. In the regression of biological offspring BMI on rearing parental SES (i.e., the SES of their biological parents who reared them), the regression coefficient was -.42 (p = .0004). By comparison, in the regression of adopted offspring BMI on rearing parental SES, the regression coefficient was smaller in absolute value (-.25), but also statistically significant (p = .0096). To assess the difference in association for biological and adopted children, we conducted tests that contrasted coefficients within models by following the general approach described by Neter et al [26] and also with conventional interaction terms as applicable. The difference between the slopes of the regressions of offspring BMI on rearing parent SES was not significant (p = .22), but a test of whether the coefficient for adopted offspring was different than half the value of the coefficient for biological offspring (as would be predicted from the CASO results) was also not significant with a much larger p-value (p = .75). This suggests that SES and a correlation between the genetic influences on BMI and the genetic influences on SES each accounted for roughly half of the correlation between rearing environment SES and BMI.

Among biological offspring, when mid-parental BMI (the average of mother and father BMI) was added to the model, the absolute value of the coefficient fell 47%, from -.42 to -.20 (p = .10 after adjusting for mid-parental BMI; p<.0001 for the reduction in value). In contrast, for the adopted offspring, when the mid-parental BMI of rearing parents was added to the model, the coefficient was virtually unchanged, from -.25 to -.22 (p = .09 for the change), and remained statistically significant (p = 0.02). In other words, the effect of SES was reduced in the presence of mid-parental BMI for biological children but was not significantly reduced for adopted children. After adjusting for mid-parental BMI, the apparent effect of rearing-parent SES was essentially the same in biological and adopted children (see Figure 2, panels C & D).

## Discussion

Our results, from two well-characterized adoption datasets, are consistent with a model in which both the rearing parents' SES and the genetic influence on BMI and its relation to SES contribute equally to the association between rearing parents' SES and the adiposity of their offspring.

Beyond the aforementioned publication examining the CASO study, we know of no other studies which have taken the approach we have to consider adoption as a form of ‘natural randomization’ to better assess the extent to which the SES association with obesity represents an effect of SES causing obesity. However, there is an existing literature discussing multiple alternative causal hypotheses and the fact that the nature and direction of causation is not known [16], [28]. Despite this, it is generally taken for granted that the SES obesity relationship represents causation from SES to obesity. For example, after analyzing an ordinary observational epidemiological study [29], one author wrote “There appears to be a protective effect of higher SES on the weight status of children and adolescents and it is likely that a wide range of socio-cultural factors influence the risk of obesity, including typical social determinants of health such as income, education, access to nutritious food, access to and affordability of sporting facilities, health literacy, outdoor environment, and cultural norms of eating, exercising and ideal weight. As such, the prevention of childhood obesity is most likely to succeed if these sociocultural determinants are addressed in interventions targeting schools, communities and other areas of social and economic disadvantage.” In addition, components embedded within the environment, factors such as crime, financial hardship, violence, and parental neglect may also influence the SES-obesity association. Before we make definitive causal statements, however, we need to better assess these potential underlying causal mechanisms (something that cannot be easily accomplished). Our approach to using adoption data is one method of doing so. Another may be the use of studies randomizing persons to receive additional money. Such studies were done in the 1960s and 1970s, [30] though we are unaware of any which specifically assessed effects on weight, BMI, or obesity (except for birth weight). Ironically, even in present time people are randomized to have more or less money every day by casinos and lotteries and acquiring data on persons who participate may be natural ways to further attempt to assess causation. There is also importance in assessing fundamental mechanisms of causation. This includes the mechanisms by which obesity may lead to lower SES, the common genetic underpinnings, and the mechanisms by which SES may cause obesity.

With respect to obesity potentially leading to lower SES, obesity appears to reduce wages given equal qualifications [31], reduce the likelihood of being hired even given equal qualifications [32], reduce the probability of attending college even given equal qualifications [33], predispose toward marrying men of lower SES among women [34], and may impair cognitive functioning and health over many years leading to reduced earning capacity [35]. With respect to the potential effects of SES on obesity, common thinking is that the economic aspects per se (e.g., the relative costs of various foods) are driving factors [36]. However, we hypothesize that the ‘socio’ as much or more than the ‘economic’ in socioeconomic status may cause the connection to obesity. That is, the self-perception of being low in a social hierarchy, apart from any specific economic factors, may lead to physiologic, cognitive, and behavioral changes that ultimately result in the anatomical changes we call obesity. As evidence of this, consider that subordinate status birds across many species (willow tit, great tit, greenfinch, chickadees, titmouse, nuthatch) carry greater fat reserves than dominant status birds [37], [38], subordinate status rats are hyperphagic and gain more fat mass when removed from dominant status rats, subordinate hamsters and monkeys consume more and increase body weight during hierarchical interactions [39]–[41] and in humans, lower subjective social status appears to be associated with higher waist to hip ratio and BMI levels to a greater degree than are objective economic indicators [42]. Finally, with respect to ideas about common mechanisms underlying SES and obesity, consider the work of Chib et al. [43] who found reciprocal relations between “…the incentive value of food and of money” in several experiments in which the hunger levels of human subjects were manipulated suggesting connections between the biological mechanisms of drives for money and food as has also been indicated in fMRI research.

Our study has several strengths. First, the study made new use of adoption data to address a set of important questions that could not be ethically investigated via a randomized experimental trial (c.f., [44]). Second, we used data from two large, well-characterized datasets related to voluntary adoption. In both populations, it appears reasonable to assume that the SES of the rearing environment was independent of the genetic predisposition to adiposity. Third, we used thorough statistical analyses permitting us to tease out the associations of interest while controlling for covariates and other sources of potential bias. Fourth, despite differences in the time the data were collected, the country of origin, and the race/ethnicity of the samples, the results were remarkably consistent. This consistency suggests that the findings are durable and supports the validity of the causal inferences we made from the observed associations.

The limitations of this study include: the datasets relied upon self-reported height and weight as opposed to direct measurements, and moreover, some of the parental weight and height data were derived by proxy self-report by the children; the age at which adoptee BMI was assessed was quite different in the two studies; the two populations studied had relatively low rates of obesity; certain biases may have been introduced by the use of mail questionnaires; the adoptees in the CASO study did not come from abroad, raising the possibility that some adoptees were, in fact, familial adoptions (however, while we cannot eliminate this possibility entirely, the Danish Adoption Register sought to filter out all adoptions where there was some relationship between the child and the adoptive parents); and we did not investigate the role additional variables such as neighborhood factors (e.g., stress, crime, violence) might have had upon the results.

Given these limitations, despite the fact that the two study populations we analyzed samples from were quite different (e.g., HOLT study parents were better educated, offspring BMI was measured in the CASO study many years after they left their rearing environment), it should be examined whether our finding holds in other groups and time periods because the relation between SES and obesity does not appear to be constant across populations or within populations across time [3], [4]. Hence, reassessing with more recent data may be valuable, especially if obesity is more prevalent, although the shorter the follow-up, the more limited the ability to study long-term effects.

In comparison with the previous analyses conducted with CASO [21], the present study adds important evidence elucidating the nature of the association between SES and adiposity. First and foremost, the inverse correlation between adoptive parental SES and offspring BMI was confirmed in independent and in very different study populations. Second, the present study demonstrated that this correlation was essentially independent of the BMI of the adoptive parents, which was only indirectly inferred in the previous study. Third, the present study provided consistent estimates of the contribution of the established genetic parent-offspring correlation in BMI to the observed inverse correlation between parental SES and offspring BMI in natural families in which the biological parents rear their own biological offspring.

The finding that the association between the SES of the rearing environment and offspring adiposity has a component that is independent of the BMI of the rearing parents strongly suggests that the mechanism is not to be found in the frame of what is considered ‘cultural transmission’ of obesogenic factors in the family environment. It is possible that the SES or related psychosocial factors (e.g., cognition and educational proficiency) of the offspring are mediating the effects of parental SES by being related to both the SES of the rearing parents and the subsequent development of adiposity in the offspring [9], [11], [12], [19]. In the former analyses of CASO, the inclusion of the SES of the adoptees in the analysis only partly reduced the correlation between parental SES and adoptee BMI [21]. Assessment of the association between SES of the rearing environment and BMI of the adoptees in childhood, when the parent-offspring correlations in BMI are established [16], would allow estimation of influences that are not driven by the SES of the adoptees themselves. The SES of the rearing environment may also be a proxy for more specific environmental or psychosocial factors that contribute to adiposity such as those that under extreme conditions may make parental neglect of their offspring a very strong predictor of later development of obesity [45]. Future research should seek to identify those factors that are causal and may be amendable and to assess if and how they may interact with the genetic predisposition to obesity [46], [47].

In conclusion, across two different datasets collected during two different time periods, in two different countries, and for two different ethnicities, we found remarkably similar results. These results suggest that roughly half of the association between the SES of the rearing parents and the subsequent BMI of their biological offspring whom they rear is due to a potential causal influence of the rearing parents' SES and that roughly half is an association due to a genetic correlation between BMI and SES. On the one hand, implication of some degree of causation is positive because it suggests that if we can identify the specific aspects of low SES that predispose to obesity, we may be able to influence such factors to achieve reductions in obesity risk. On the other hand, the results suggest that the effects of any such manipulations should be expected to, at most, have an effect equivalent to half of that which would be expected if the association were all causal.

## Supporting Information

Appendix S1 **Simulation Studies.**
(DOC)
