# Supplementary material for: Is Socioeconomic Status of the Rearing Environment Causally Related to Obesity in the Offspring?
Source: PLoS One. 2011 Nov 16;6(11):e27692. doi: 10.1371/journal.pone.0027692 (PMC3218016; doi:10.1371/journal.pone.0027692)
Supplement: Appendix S1 — Simulation Studies. (DOC) [file pone.0027692.s001.doc]

**Simulation Studies**

In principle, data set inclusion criterion #5 could have introduced some bias. In practice, whether any such bias may be large or trivial will depend on the nature of the population correlation structure among the variables studied, the sample sizes available, and the number of samples which had to pass this inclusion criterion. Although we had a very strong intuition that any bias intruded would be trivial based on the large sample sizes available and the magnitudes of correlations observed, we conducted computer simulations to evaluate this. In these simulations, we used the correlation structure implied in the path diagram depicted in the accompanying Figure.


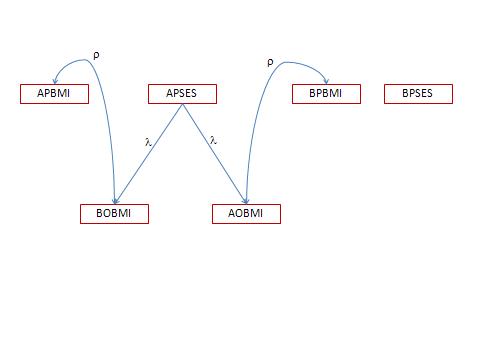
The six variables: AP, BP, BO, and AO denote adopting parents, biological parents, biological offspring, and adopted offspring, respectively. Rho and lambda represent correlations and slopes (or causal effects), respectively. Without loss of generality, all variables were set to have unit variance and mean zero. Using this structure, we generated data from a multivariate normal distribution with rho set to .30 and lambda set to -.20 to approximate the values in our observed data under a model in which all of the Parental SES-Offspring BMI association was causal. This might be considered a ‘null model’ for us. We simulated 1,000 dataset trios where, within each trio there were three datasets, one having the size of the Colorado dataset, one having the size of the Holt dataset, and one having the size of the CASO dataset. For each dataset within each trio, we regressed BOBMI on APSES separately in each dataset. We then retained within each trio for which the sample slope was both negative in sign and statistically significant. Otherwise, the dataset was discarded. Then, for each dataset retained (i.e., excluding the discarded datasets), we calculated the difference between the slope of BOBMI regressed on APSES and the slope of AOBMI regressed on APSES and denoted this difference delta_1 and also calculated the difference between the slope of AOBMI regressed on APSES and the slope of AOBMI regressed on BPSES and denoted this difference delta_2. If there is no bias introduced by our selection method, then the expected values for delta_1 and delta_2 are zero and .20, respectively.

As can be seen in the Table below, there was no detectable bias. This is because the power to detect the negative slope on which the dataset inclusion criterion was based was close to 1.0.

Sensitivity analyses involving the use of bootstrap yielded consistent results (data not shown), thus suggesting no concerns about the non-normality of the residual distribution influences the results. The bootstrap sampling was implemented using SPSS’ non-linear regression procedure. Bootstrap samples were taken with replacement as is standard practice. One thousand bootstrap samples were used. The standard deviation of the parameter estimates across the 1,000 bootstrap samples was taken to be the bootstrap estimate of the standard error of the parameter estimate. Non-parametric asymptotic confidence intervals were constructed using this SE estimate. Moreover, after transformation, the residual distribution closely approximated a normal distribution.

| **Simulation Results** | | | |
| --- | --- | --- | --- |
| **Holt Study** | | | |
| Variable | N | Mean | SD |
| delta1 | 1000 | 0.000 | 0.035 |
| delta2 | 1000 | 0.201 | 0.034 |
|  |  |  |  |
| **Copenhagen Study** | | | |
| Variable | N | Mean | SD |
| delta1 | 1000 | -0.003 | 0.050 |
| delta2 | 1000 | 0.198 | 0.049 |
|  |  |  |  |
| **Colorado Study** | | | |
| Variable | N | Mean | SD |
| delta1 | 889 | -0.012 | 0.084 |
| delta2 | 889 | 0.200 | 0.091 |

**Handling Missing Data**

| **Imputed Variables** | |
| --- | --- |
| **Copenhagen Study** | |
| **Variable** | **% Missing** |
| Parental SES | 4.0 |
| Mid-parental BMI | 11.3 |
| Child's BMI | 5.8 |
| **Holt Study** | |
| **Variable** | **% Missing** |
| Birth Father's SES | 23.6 |
| Adoptive Father's SES | 3.5 |
| Birth Mother's BMI | 36.5 |
| Birth Father's BMI | 48.7 |
| Adoptive Mother's BMI | 26.1 |
| Adoptive Father's BMI | 33.8 |
| Adoptee's BMI | 14.9 |

Missing data is a potential problem in any study on which it occurs. Although the most common approach in epidemiological studies to missing data is to use ‘listwise deletion’, that is to only include cases with complete data, it is well-established in the statistical literature that doing so is sub-optimal, both lessening power and potentially creating biases. Instead, we used multiple imputation (MI). Although MI is not a panacea, nor is any method of handling missing data, it is preserves power, makes full use of the available data, is dependent on fewer assumptions than is listwise deletion and therefore valid under a broader range of conditions than is listwise deletion [1]. The list of imputed variables and their missingness is provided in the Table to the right. For each real data set (Holt and CASO), point estimates and estimated variances were computed in each of 5 imputed data sets. Then, for each real data set, combined and significance tests were conducted as described by Schafer [2]. For multiple degree of freedom tests, p-values from the separately imputed datasets were combined as described by Shaffer (2, page 115, equations 4.39 to 4.41).

**CASO Data Set**: For the CASO data set, we did not want to rely on the normality assumption in the imputation step, because of the original sampling procedure used to increase statistical efficiency (i.e., power per person studied) resulted in a markedly non-normal distribution of BMI variables. Hence, in SPSS MVA (missing value analysis) procedure, we used the multiple imputation procedure and created imputed values by adding residuals sampled from the empirical distribution to the predicted values from a regression model [2, 3].

**Holt Data Set**: Missing values were imputed via Proc MI in SAS, creating 5 sets of imputed values. We used the default for Proc MI, specifically, the Markov Chain Monte Carlo (MCMC) method with a single chain to create five imputations. The posterior mode, the highest observed-data posterior density, with a non-informative prior, is computed from the EM algorithm and is used as the starting value for the chain [4].

**References**

1. Allison PD. Multiple imputation for missing data. Sociological Methods & Research. 2000; 28: 301-309.
2. Schafer JL. Multiple imputation: a primer. Stat Methods Med Res. 1999;8:3-15.
3. [http://support.spss.com/ProductsExt/SPSS/ESD/17/Download/User%20Manuals/English/SPSS%20Missing%20Values%2017.0.pdf](http://support.spss.com/ProductsExt/SPSS/ESD/17/Download/User Manuals/English/SPSS Missing Values 17.0.pdf)
4. SAS/STAT 9.1 User's Guide, SAS Institute, SAS Publishing Staff, SAS Publishing, 2004.

**Clarification of A Key Expectation**

Regarding the sentence on page 11, “This suggests that the possible causal effect of the SES of the rearing environment and a correlation between the genetic influence on BMI and its influence on SES each accounted for roughly half the correlation between rearing environment SES and BMI,” this conclusion is drawn on the basis of the prior sentence which states “The difference between the regression coefficients for the biological and the adoptive father’s SES was not significant (p=0.982) and the two coefficients were very similar (-.22 and -.23, respectively).” The rationale for this can be seen from the expanded path diagram below.


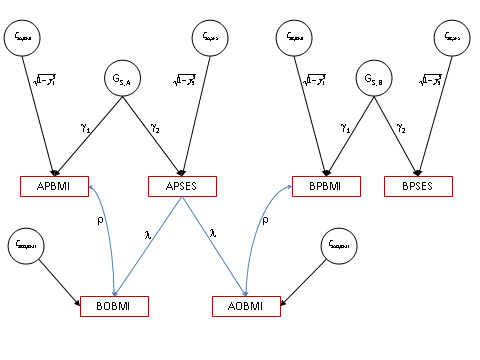


This diagram portrays a model which is expanded from the simplified null model offered earlier in response to point #2. This diagram follows the established conventions of path diagrams as originally put forth by Wright [1, 2], and thoroughly described elsewhere [3]. Single-headed arrows represent direct causal effects, double headed curved arrows represent correlations without a specification of the causal origin, variables in rectangles are observed variables as defined earlier, and variables in circles are latent unobserved variables. Without loss of generality, all variables are set to have mean zero and variance one, and symbols adjacent to lines with arrows are coefficients which can take values on the range [-1, 1]. G_S,A_ and G_S,B_ are, respectively, latent genetic factors which affect both SES and BMI, the various ζ variables are all other exogenous factors which affect BMI and SES.

Under such a model, we can mathematically express the three quantities referenced in the two sentences quoted above.

The correlation between the SES of the rearing parental environment and the BMI of the parents’ biological offspring who are being reared in that environment = $\rho_{APSES,BOBMI}=\lambda+\rho\gamma_{1}\gamma_{2}$. The correlation between the SES of the rearing parental environment and the BMI of the adopted offspring $\rho_{APSES,AOBMI}=\lambda$. Finally, the correlation between the SES of the biological parents and the BMI of their adopted-away biological offspring who are being reared by the adoptive parents = $\rho_{BPSES,AOBMI}=\rho\gamma_{1}\gamma_{2}$. From there it is easy to see that under this model, the first correlation, generally the association measure being estimated when people are talking about the association between SES and BMI among children, is composed of two components, which can be seen to be the other two correlations or association measures. Hence, observing that the latter association measures are equal to each other and are also half the size of the first one is evidence consistent with the model and in which the usual SES-BMI association is contributed to equally by a direct causal effect and a shared common genetic diathesis.

**References**

Wright S. On the Nature of Size Factors. Genetics. 1918; 3: 367-74.

Wright S. The Method of Path Coefficients. Annals of Mathematical Statistics. 1934; 5: 161-215.

1. Boker SM, Neale MC. McArdle JJ. An algorithm for the hierarchical organization of path diagrams and calculation of components of expected covariance. Structural Equation Modeling. 2002; 9: 174-194.
